# Coronary Artery Computed Tomography Angiography for Preventing Cardio-Cerebrovascular Disease: Observational Cohort Study Using the Observational Health Data Sciences and Informatics’ Common Data Model

**DOI:** 10.2196/41503

**Published:** 2022-10-13

**Authors:** Woo Kyung Bae, Jihoon Cho, Seok Kim, Borham Kim, Hyunyoung Baek, Wongeun Song, Sooyoung Yoo

**Affiliations:** 1 Department of Family Medicine, Health Promotion Center Seoul National University Bundang Hospital Republic of Korea Bundang-gu, Seongnam-si, Gyeonggi-do Republic of Korea; 2 Healthcare Information and Communication Technology Research Center, Office of eHealth Research and Business Seoul National University Bundang Hospital Republic of Korea Seongnam-si Republic of Korea

**Keywords:** cardiovascular diseases, coronary artery computed tomography angiography, observational study, common data model, population level estimation, cardiology, vascular disease, medical informatics, computed tomography, angiography, electronic health record, risk score, health data science, data modeling

## Abstract

**Background:**

Cardio-cerebrovascular diseases (CVDs) result in 17.5 million deaths annually worldwide, accounting for 46.2% of noncommunicable causes of death, and are the leading cause of death, followed by cancer, respiratory disease, and diabetes mellitus. Coronary artery computed tomography angiography (CCTA), which detects calcification in the coronary arteries, can be used to detect asymptomatic but serious vascular disease. It allows for noninvasive and quick testing despite involving radiation exposure.

**Objective:**

The objective of our study was to investigate the effectiveness of CCTA screening on CVD outcomes by using the Observational Health Data Sciences and Informatics’ Observational Medical Outcomes Partnership Common Data Model (OMOP-CDM) data and the population-level estimation method.

**Methods:**

Using electronic health record–based OMOP-CDM data, including health questionnaire responses, adults (aged 30-74 years) without a history of CVD were selected, and 5-year CVD outcomes were compared between patients undergoing CCTA (target group) and a comparison group via 1:1 propensity score matching. Participants were stratified into low-risk and high-risk groups based on the American College of Cardiology/American Heart Association atherosclerotic cardiovascular disease (ASCVD) risk score and Framingham risk score (FRS) for subgroup analyses.

**Results:**

The 2-year and 5-year risk scores were compared as secondary outcomes between the two groups. In total, 8787 participants were included in both the target group and comparison group. No significant differences (calibration *P*=.37) were found between the hazard ratios of the groups at 5 years. The subgroup analysis also revealed no significant differences between the ASCVD risk scores and FRSs of the groups at 5 years (ASCVD risk score: *P*=.97; FRS: *P*=.85). However, the CCTA group showed a significantly lower increase in risk scores at 2 years (ASCVD risk score: *P*=.03; FRS: *P*=.02).

**Conclusions:**

Although we could not confirm a significant difference in the preventive effects of CCTA screening for CVDs over a long period of 5 years, it may have a beneficial effect on risk score management over 2 years.

## Introduction

Cardio-cerebrovascular diseases (CVDs) result in 17.5 million deaths annually worldwide, accounting for 46.2% of noncommunicable causes of death, and are the leading cause of death, followed by cancer, respiratory disease, and diabetes mellitus [1]. CVDs involve demographic factors (age, sex, and family history), pre-existing conditions (hypertension, diabetes mellitus, and hyperlipidemia), and lifestyle and environmental factors. Unlike demographic characteristics, lifestyle factors, such as an inappropriate diet, a lack of exercise, smoking, stress, and excessive drinking, can be improved to reduce the risk of CVDs [2].

Coronary artery computed tomography angiography (CCTA) detects calcification in the coronary arteries and can be used to detect asymptomatic but serious vascular disease. It allows for noninvasive and quick testing despite involving radiation exposure [3,4]. For these reasons, many studies have investigated the early detection of CVDs by using CCTA, which enables prompt treatment and results in better outcomes.

In recent years, there has been debate about whether screening via CCTA helps prevent CVDs in populations with varying degrees of risk. CCTA has been recommended to predict CVDs in patients with cancer [2,5], but among asymptomatic individuals, the evidence about its effectiveness is inconsistent.

We aimed to study the effectiveness of CCTA screening by analyzing observational health checkup data from electronic health records (EHRs) in the form of the Observational Medical Outcomes Partnership Common Data Model (OMOP-CDM), using a cohort study design [6]. The OMOP-CDM standardizes disparate data and enables the analysis of deidentified, large-scale observational data in a distributed research data network. Moreover, as the data are standardized, the same analytical codes can be used to conduct efficient analyses through the data network. Observational Health Data Sciences and Informatics (OHDSI)—an open international collaborative community—provides an open-source analytics tool for OMOP-CDM data that produces scientific, reliable, and reproducible evidence.

Using the OHDSI analytics tool, we performed a comparative effectiveness study of CVD outcomes in asymptomatic patients without a history of CVD who underwent a health checkup at a tertiary university hospital. The conventional assessments of CVD risk, namely assessments of the Framingham risk score (FRS) and the American College of Cardiology/American Heart Association (ACC/AHA) atherosclerotic cardiovascular disease (ASCVD) risk score, were used to stratify the participants into high-risk and low-risk groups for stratified analyses. Although the risk of CVD increases with age, we compared differences between the two groups after 2 and 5 years to assess the short-term benefits of CCTA-based screening and whether it can help prevent CVDs.

## Methods

### Data Sources

The study site was the Seoul National University Bundang Hospital (SNUBH), which is located in the Seoul metropolitan area. The SNUBH collected OMOP-CDM version 5.3 data based on EHRs from 2003 to 2020. The data included patients’ demographic information, clinical information (diagnoses, medications, tests, surgeries and procedures, family histories, past histories, and nursing flowcharts), and health questionnaire responses. The health questionnaire responses about medical history, family history, socioeconomic status, medication history, marital status, exercise and physical activity status, and depression assessment results were converted to OMOP-CDM data. In this study, we used the deidentified OMOP-CDM data that the SNUBH collected from over 2 million patients, including outpatients, inpatients, and emergency department visits.

### Ethical Considerations

This study adhered to the relevant guidelines and regulations of the SNUBH Institutional Review Board (IRB). As the OMOP-CDM is a deidentified data set, the study was exempted from review by the SNUBH IRB (IRB number: X-2202-736-903).

### Study Design

This was a retrospective, observational, comparative cohort study that used OMOP-CDM–formatted EHR data. We analyzed data from adults aged 30 to 74 years who underwent a health checkup between April 1, 2003, and December 31, 2015, and were followed up for at least 5 years. Only those who responded to the questionnaire item about medical history in the health checkup survey were included. Individuals with a history of CVD were excluded from this study. The index date was set as the date of completing the health checkup questionnaire at a health checkup visit for the first time. CVDs that occurred within 60 days of the index date were considered as cases in which patients were diagnosed during the health checkup, and these CVD events were excluded as CVD outcomes. Thus, the outcome was defined as CVD events that occurred 60 days after the index date, and follow-ups ended on the date that CVD events occurred (ie, within 5 years from the index date), the date of the final hospital visit, or the date of death. As such, the time-at-risk period was set as 61 days after the index date to 5 years after the index date.

The primary outcome was the comparison of CVD hazard ratios (HRs) between the group that underwent CCTA (target group) and the group that did not undergo CCTA at the health checkup visit (comparison group).

In the subgroup analyses, the CVD HRs, which were based on the ACC/AHA ASCVD risk score and the FRS, were analyzed. The patients were stratified into the nonrisk and low-risk group or the high-risk group based on a cutoff score of 10 for the FRS [7] and 5 for the ASCVD risk score [8].

The secondary outcome was the difference between the risk scores of patients who underwent health checkups 2 years and 5 years after the index date. The differences between the risk scores at the index date and those at the times of subsequent examinations were used for comparative analyses.

### Study Population

From April 2003 to December 2015, a total of 69,334 patients aged 30 to 74 years were enrolled for a health checkup. Of these patients, only 49,496 responded to the questionnaire, and only 46,087 patients had no cardiovascular history. A total of 42,489 patients for whom we could calculate the risk score—a key indicator of this study—were selected as the initial cohort.

**Figure 1:**
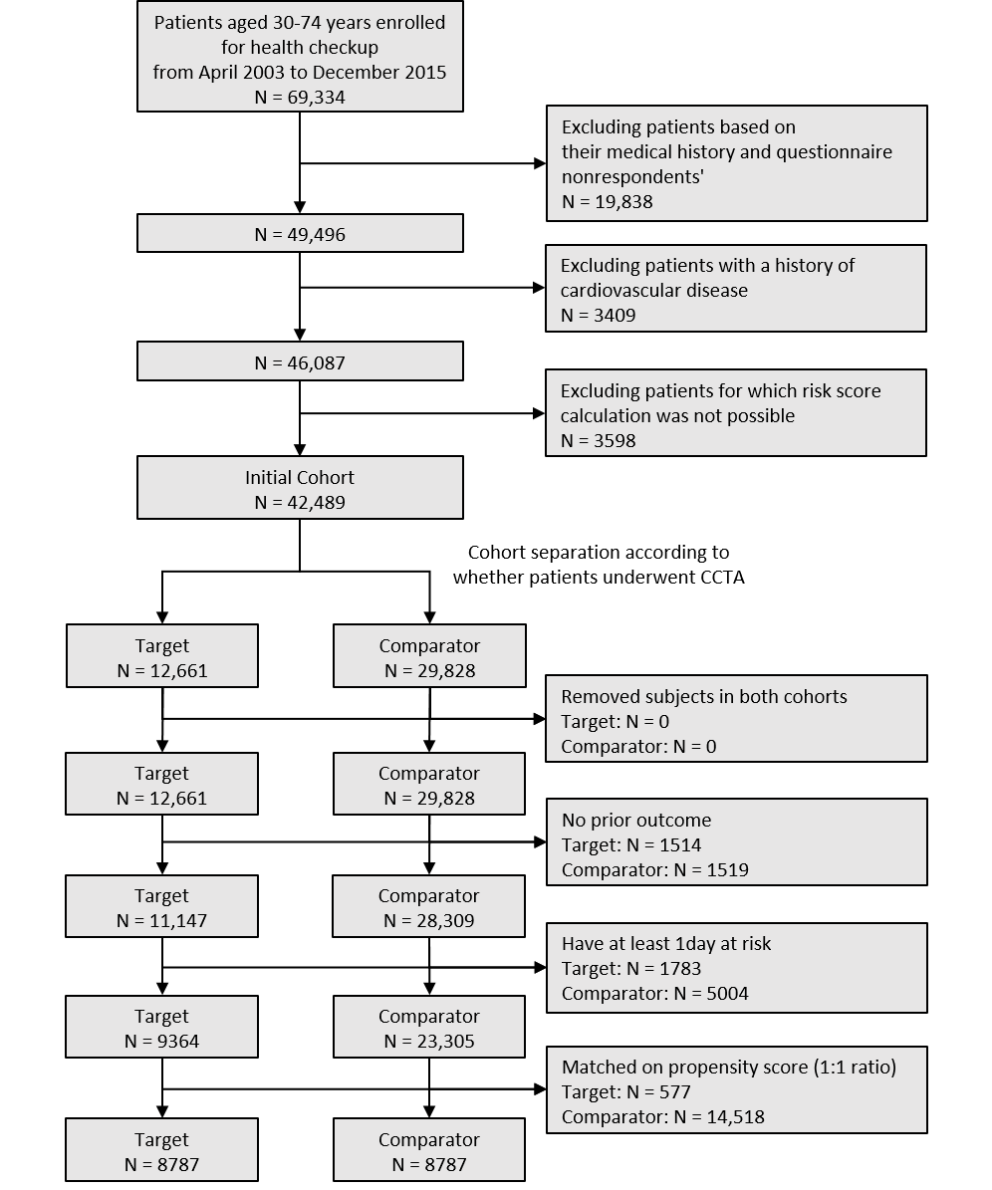
The flowchart of the study population. CCTA: coronary artery computed tomography angiography.

Initially, of the 42,489 patients who were included in the analysis, 12,661 underwent CCTA (target group), and 29,828 did not (comparison group). Of these patients, 1514 from the target group and 1519 from the comparison group with a history of CVD before the index date were excluded from the analysis. In addition, 1783 patients from the target group and 5004 patients from the comparison group who did not fulfill the minimum observation period of 1 day during the time-at-risk window were excluded. The remaining 9364 patients from the target group and 23,305 patients from the comparator group underwent 1:1 propensity score matching. During 1:1 propensity score matching, 577 people who did not match the comparator group were excluded from the target group because matching was performed to maximize the minority group, and 14,518 people were excluded from the comparator group. Finally, 8787 of the 12,661 patients (69.4%) from the initial target cohort were selected as the final target group, and 8787 of the 29,828 patients (29.5%) from the initial comparator cohort were used for the analysis as the final comparator group (Figure 1).

### Covariates

Approximately 13,000 variables were used as covariates for propensity score matching. These covariates included patient clinical data that were obtained at any time prior to the index date and health checkup data that were obtained on the index date. The patient clinical covariates included the condition era, the condition group era, the drug group era, observations, measurements, procedures, the Charlson Comorbidity Index score, the Diabetes Complications Severity Index score, the CHADS_2_ (Congestive Heart Failure, Hypertension, Age, Diabetes, Previous Stroke/Transient Ischemic Attack [2 points]) score, the CHA_2_DS_2_-VASc (Congestive Heart Failure, Hypertension, Age≥75 [Doubled], Diabetes, Stroke [Doubled], Vascular Disease, Age 65 to 74, and Sex Category [Female]) score, and the hospital frailty risk score. The covariates that were measured at the index date included demographic data, such as sex, age, education level, average monthly income, and marital status; health questionnaire data, such as any history of cancer and chronic diseases (hypertension, diabetes, and hyperlipidemia), medication history (antihypertensive drugs, antidiabetic drugs, antihyperlipidemic drugs, and aspirin), smoking status, and family history; and health checkup data, such as height, weight, BMI, blood pressure (systolic and diastolic), waist circumference, glucose levels, uric acid levels, aspartate aminotransferase levels, alanine aminotransferase levels, triglyceride levels, total cholesterol, high-density lipoprotein cholesterol levels, low-density lipoprotein cholesterol levels, and glycated hemoglobin A1c levels.

### Outcomes

The outcome of this study was the first registered CVD event, which was based on a CVD diagnosis during the observation period. A CVD event was defined based on *International Classification of Diseases, 10th Revision* (ICD-10) codes I20 to I25 (ischemic heart disease), I50 (heart failure), I60 to I69 and G45 to G46 (stroke), and E78 (hypercholesterolemia). As we intended to assess the HRs of CVDs resulting from arteriosclerotic diseases only, we excluded cardiogenic diseases, such as atrial fibrillation and aneurysm (I42-I43, I48, I71, I62, and I68), and diseases caused by external accidental factors (I60 and I62). The ICD-10 codes that were chosen as the outcomes were reviewed by 1 clinical specialist and 1 nurse.

### Statistical Analysis

We used the population-level estimation methodology and an open-source tool provided by OHDSI [9]. All analyses were performed by using R version 4.0.3 (R Foundation for Statistical Computing) [10]. Large-scale propensity score matching [11] was performed to adjust for potential confounding and to resolve the imbalance between the target and comparison cohorts caused by selection bias—a result of the retrospective observational nature of this study. The propensity score–matched model, which used approximately 13,000 covariates, was fitted through regularized regression, and the propensity score was calculated as the probability of a patient undergoing CCTA based on the covariates. Target and comparison group patients with similar propensity scores were matched to create a balanced cohort. To establish a matched cohort, we performed 1:1 propensity score matching by using a caliper width of 0.2 of the SD of the logit. The conditional Cox proportional hazards model was used to estimate HRs for the target group, in relation to the comparison group. The balance of the covariates between the cohorts was assessed based on the standardized difference of the mean (<0.1). Statistical significance was evaluated at *P*<.05 for 2-tailed tests.

To explain any residual bias after controlling for the measured covariates, we used negative control outcomes that were unlikely to be induced or prevented by undergoing CCTA; thus, the actual HR was anticipated to be 1. The negative control outcomes were selected by a clinical specialist through a manual review of the outcomes that were used in a previous OHDSI study [12] (Table S1 in Multimedia Appendix 1). The same study design was used to estimate the outcomes of interest and calculate the HR estimate for the negative control group, and all HR estimates were presented with 95% CIs and *P* values, along with the empirical null distribution and adjustment [13,14]. The empirical equivalence of the two cohorts was assessed by using the propensity score distribution. We also reported the power analysis; propensity score; cohort balance before and after propensity score matching; fitted null distribution; calibration chart for negative control outcomes; and Kaplan-Meier curve, which shows the proportional hazards assumption over time.

To confirm the changes in the differences in ASCVD risk scores and FRSs, we used the 2-group comparison method. The normality of the amount of change was confirmed by using the Shapiro-Wilk test, and the changes in the two groups were confirmed by using the Wilcoxon rank-sum test.

## Results

### Characteristics of Study Participants

**Figure 2:**
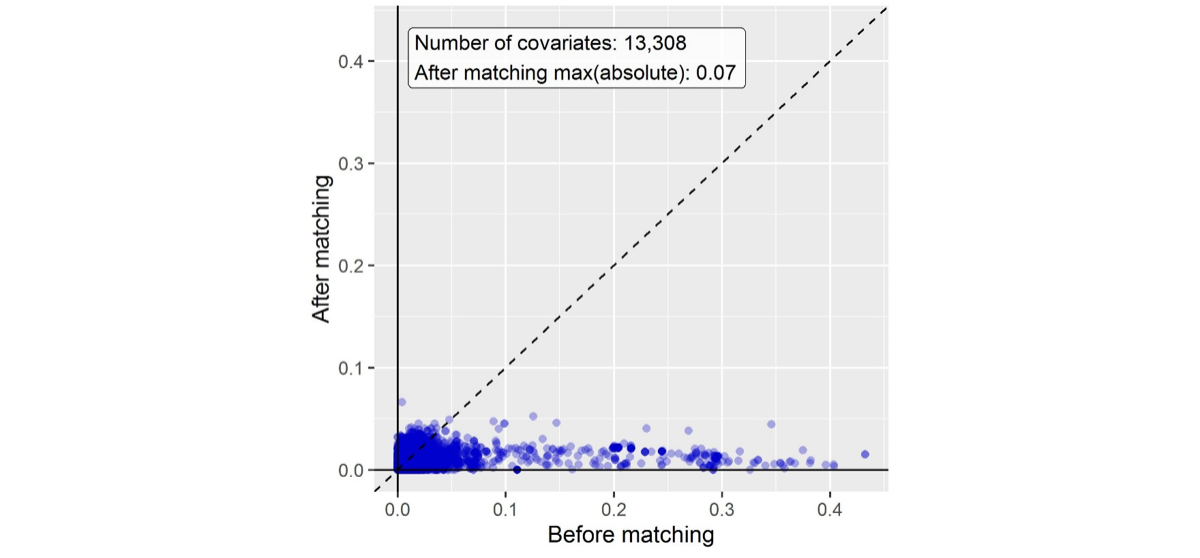
Standardized difference of means between the two groups of covariates before and after propensity score matching.

**Figure 3:**
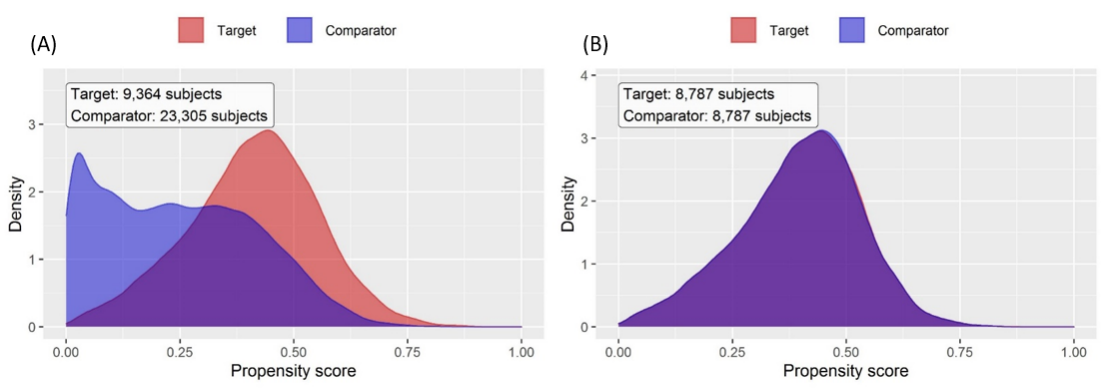
Distribution of propensity scores in each group (A) before and (B) after propensity score matching.

Table 1 shows the baseline characteristics of the patients before and after propensity score matching. The table shows the patients’ age groups, sex, and BMIs; the number of patients in the risk score groups; and the follow-up periods. For most demographic characteristics, the differences between groups decreased after matching. The standardized difference of the mean for the covariates decreased from 0.4 to 0.07 after propensity score matching, which is lower than the conventional standard of 0.1, thereby confirming that propensity score matching was performed correctly (Figure 2). This can also be observed in Figure 3, which compares the distributions from before and after propensity score matching.

**Table 1:** The baseline characteristics of the study population before and after propensity score matching.

Characteristics			Before matching							After matching				
			CCTA^a^ group (n=12,661)		Non-CCTA group (n=29,828)		Standard difference		CCTA group (n=8787)			Non-CCTA group (n=8787)		Standard difference
**Age group^b^ (years), n (%)**														
	30-34	226 (1.8)		2442 (8.2)		−0.26		150 (1.7)			155 (1.8)		0	
	35-39	1043 (8.2)		4319 (14.5)		−0.19		761 (8.7)			700 (8)		0.03	
	40-44	1870 (14.8)		5257 (17.6)		−0.08		1406 (16)			1263 (14.4)		0.05	
	45-49	2516 (19.9)		5134 (17.2)		0.07		1846 (21)			1697 (19.3)		0.04	
	50-54	2435 (19.2)		4617 (15.5)		0.10		1702 (19.4)			1678 (19.1)		0.01	
	55-59	2084 (16.5)		3322 (11.1)		0.16		1373 (15.6)			1438 (16.4)		−0.02	
	60-64	1468 (11.6)		2275 (7.6)		0.14		908 (10.3)			1050 (11.9)		−0.05	
	65-69	734 (5.8)		1564 (5.2)		0.02		471 (5.4)			568 (6.5)		−0.05	
	70-74	285 (2.3)		898 (3.0)		−0.05		170 (1.9)			238 (2.7)		−0.05	
**Sex^b^, n (%)**														
	Female	4757 (37.6)		12,650 (42.4)		−0.10		3561 (40.5)			3368 (38.3)		0.04	
	Male	7904 (62.4)		17,178 (57.6)		0.10		5226 (59.5)			5419 (61.7)		−0.04	
BMI^b^ (kg/m^2^), mean (SD)			24.2 (3.1)		23.7 (0.2)		0.18		24.0 (3.1)			24.1 (3.1)		−0.03
**ACC/AHA^c^ ASCVD^d^ risk score^e^, n (%)**														
	High (≥5)	5036 (39.8)		8576 (28.8)		N/A^f^		3062 (34.8)			3493 (39.8)		N/A	
	Low (<5)	7625 (60.2)		21,252 (71.2)		N/A		5725 (65.2)			5294 (60.2)		N/A	
**Framingham risk score^e^, n (%)**														
	High (≥10)	4996 (39.5)		8155 (27.3)		N/A		3030 (34.5)			3381 (38.5)		N/A	
	Low (<10)	7665 (60.5)		21,673 (72.7)		N/A		5757 (65.5)			5406 (61.5)		N/A	
Follow-up period (days)^e^, mean (SD)			2220.3 (1731.6)		1928.9 (1675.5)		N/A		2604 (1594.4)			2583.1 (1657.0)		N/A

^a^CCTA: coronary artery computed tomography angiography.

^b^Variables used in propensity score matching.

^c^ACC/AHA: American College of Cardiology/American Heart Association.

^d^ASCVD: atherosclerotic cardiovascular disease.

^e^Variables not used in propensity score matching.

^f^N/A: not applicable.

### Effect of CCTA on CVDs

**Figure 4:**
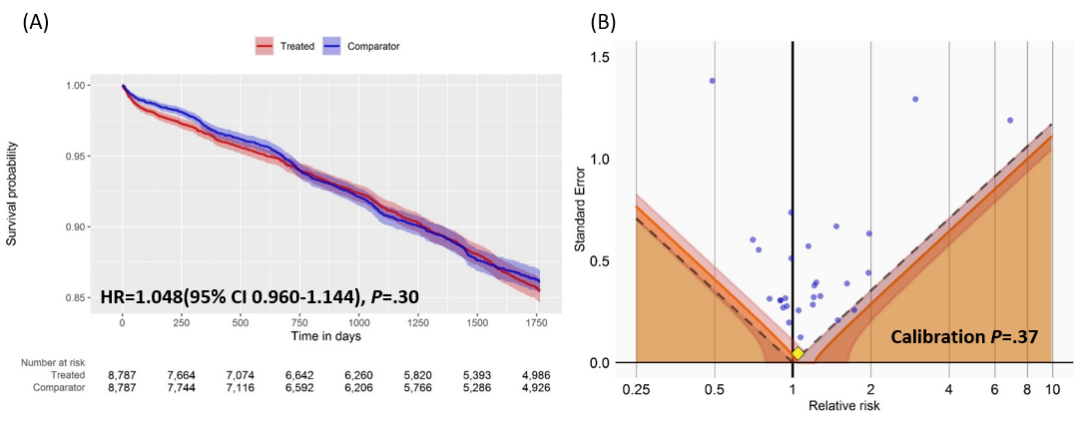
(A) Kaplan-Meier curve plot and (B) rejection area plot with negative outcome controls applied in the main analysis. HR: hazard ratio.

The Cox proportional hazards model was used to estimate and compare the HRs of CVDs among the target and comparison groups after propensity score matching, and no statistically significant differences were found between the two groups. The Kaplan-Meier analysis revealed that the HR was 1.048 (95% CI 0.960-1.144), which was not statistically significant (*P*=.30). The calibration *P* value, which was adjusted by using a negative control and was the most important indicator in our analysis, was .37, indicating no statistical significance (Figure 4).

### Subgroup Analysis

The study population was stratified based on the cutoff scores for the ACC/AHA ASCVD risk score and FRS for subgroup analyses. Table 2 presents the results of each analysis. In each subgroup, the standardized difference of the mean dropped to <0.1 after propensity score matching. Figure S1 in Multimedia Appendix 1 shows the propensity score distributions, and Figure S2 in Multimedia Appendix 1 shows the standardized difference of the mean among groups of covariates before and after propensity score matching.

In the ASCVD high-risk subgroup (risk score≥5), 3149 patients were included in both the target group and comparison group. In the low-risk subgroup (risk score<5), 5524 patients were included in both the target group and comparison group. In the high-risk and low-risk subgroups, the calibration *P* value, which was adjusted by using negative controls, was .39 and .50, respectively, showing no significant differences in the HRs of CVDs among the target and comparison groups.

In the FRS high-risk subgroup (FRS≥10), 3110 participants were included in both the target group and comparison group. In the low-risk subgroup (FRS<10), 5602 patients were included in both the target group and comparison group. The calibration *P* value, which was adjusted by using negative controls, was .13 and .57 in the high-risk and low-risk subgroups, respectively, indicating no significant differences in the HRs of CVDs among the target and comparison groups (Figure S3 in Multimedia Appendix 1).

**Table 2:** The risk of cardio-cerebrovascular disease at 5 years in each subgroup based on the American College of Cardiology/American Heart Association (ACC/AHA) atherosclerotic cardiovascular disease (ASCVD) risk score and Framingham risk score (FRS).

		Hazard ratio (95% CI)	*P* value^a^	Calibration *P* value^b^
**ACC/AHA ASCVD risk score**				
	High (≥5)	1.113 (0.984-1.259)	.09	.39
	Low (<5)	0.999 (0.881-1.133)	.99	.50
**FRS**				
	High (≥10)	1.166 (1.031-1.321)	.02	.13
	Low (<10)	1.004 (0.883-1.141)	.96	.57

^a^Kaplan-Meier analysis *P* value.

^b^Calibration *P* value that was adjusted by using a negative control.

### Risk Scores at 2 and 5 Years

The 2-year median change in the ASCVD risk scores and the FRSs of the non-CCTA group was 0.23 and 0.60, respectively. In contrast, the ASCVD risk scores and the FRSs of the CCTA group changed by 0.17 and 0.39, respectively. There was a statistically significant difference for both risk scores, with *P* values of .03 and .02, respectively.

The 5-year median change in the ASCVD risk scores and the FRSs of the non-CCTA group was 1.06 and 1.61, respectively. In contrast, the ASCVD risk scores and the FRSs of the CCTA group changed by 1.10 and 1.66, respectively. There was no statistically significant difference for both risk scores, with *P* values of .97 and .85, respectively (Table 3).

**Table 3:** Changes in the differences in American College of Cardiology/American Heart Association (ACC/AHA) atherosclerotic cardiovascular disease (ASCVD) risk scores and Framingham risk score (FRSs) from baseline at 2 and 5 years.

		CCTA^a^ group		Non-CCTA group			*P* value^b^
		Patients, n	Change in score, median (IQR)	Patients, n	Change in score, median (IQR)		
**Differences in risk scores from baseline at 2 years**							
	ACC/AHA ASCVD risk scores	1330	0.17 (−0.16 to 1.08)	1691	0.23 (−0.10 to 1.30)	.03	
	FRSs	1330	0.39 (−0.80 to 1.96)	1691	0.60 (−0.69 to 2.26)	.02	
**Differences in risk scores from baseline at 5 years**							
	ACC/AHA ASCVD risk scores	1232	1.10 (0.08 to 1.57)	1372	1.06 (0 to 2.79)	.97	
	FRSs	1232	1.66 (0.04 to 3.92)	1372	1.61 (0.09 to 4.11)	.85	

^a^CCTA: coronary artery computed tomography angiography.

^b^Wilcoxon rank-sum test *P* value.

## Discussion

### Principal Results

From our population-level estimation study, which compared the CVD HRs of a health checkup group that was undergoing CCTA with those of a group that was not undergoing CCTA over 5 years, although some benefits were observed at 2 years, we found no significant difference (calibration *P*=.37) in the final risk of CVD events between the two groups. It seems that CCTA has no beneficial effect on CVD prevention for long periods of time.

Communication about medical examinations and examination results through counseling has been reported to improve health indicators, such as CVD risk. In the Korean national health insurance service screening program, the group that underwent cardiovascular health screening for 40-year-olds had higher rates of new hypertension, diabetes, and hyperlipidemia, whereas the incidence of CVD mortality, all-cause mortality, and major adverse cardiovascular events was lower [15]. Per the results of an analysis of the same data, the group that received counseling after the health checkup had higher motivation stages of health behavior change than those of the group that received only the checkup [16]. The smoking cessation rate was higher after 2 years when compared to that of the group who received only the checkup [17]. Engberg et al [18] reported that cardiovascular risk scores, BMIs, and serum cholesterol levels were lower in the intervention groups than those in the control group after 5 years’ worth of health screenings and consultations.

In existing studies that require lifestyle modifications, such as modifications for obesity, smoking cessation, and substance abuse, the effects of 1-time interventions or short-term interventions, interviews, and counseling tend to weaken over time. In a study that used the motivational interview technique for people with substance abuse issues, the positive effect observed at 3 months disappeared at 12 months [19], and in another study, the effect of smoking cessation treatment continued for 10 weeks and gradually slowed down at 3, 6, and 12 months [20].

Our study compared patients who did or did not undergo additional coronary computed tomography. Both groups underwent the same levels of examination and counseling, which were conducted by the cardiovascular health screening program of the national service in 1 hospital.

Smoking status, blood pressure, and blood lipid concentration, which are major factors in the FRS and ASCVD pooled cohort equations score, are closely related to lifestyle changes. Similar to previous studies, the effect of a single coronary computed tomography scan and the results of counseling decreased over time, and the differences that were observed after 2 years disappeared after 5 years.

### Limitations

This study has some limitations. First, the follow-up period was 5 years, and the risk scores were not observed for a longer period (eg, 10 years, as CVDs can last for >10 years). A follow-up study for identifying a risk score that is suitable for CVD prediction over longer periods can be conducted in the future. Second, as this was a single-center study, some of the outcomes may not be generalizable. Multicenter studies that use OHDSI data networks can provide more generalizable evidence. Third, this study included patients who visited the health promotion center multiple times; those who did not undergo CCTA at the first visit but underwent CCTA during subsequent visits were included in the comparison group. Therefore, the differences between the groups might have been attenuated. This can be avoided by conducting a prospective cohort study. Lastly, observational research that uses EHR data has the limitation that it cannot fully capture the entirety of a patient's health information [21]. This study converted EHR data into common data model data, and it has the same limitation. If the participants of this study underwent examinations and treatments outside of the hospital, there was a disadvantage that the records for these procedures were not recorded in the database. Additionally, with regard to drugs, the SNUBH common data model converted data on prescription drugs for outpatients and administration drugs for inpatients. Thus, it was not known whether the drugs ordered for the outpatients were taken on time by the patients. As such, selection bias may have occurred due to information not being recorded in the database. Although it is possible to reduce channeling bias through large-scale propensity score matching, which we used in this study, there may still be the limitation that such matching cannot reduce selection bias [22].

### Comparison With Prior Work

Waugh et al [23] conducted a systematic review and meta-analysis of 5 studies and reported that computed tomography has no benefits as a screening tool for the potential onset of CVDs. However, a closer review revealed that all 5 included studies were inappropriate in terms of their findings about the prophylactic benefits of CCTA. All of these studies investigated the association between coronary artery calcium (CAC) and the onset of CVDs or death after a specific follow-up period in patients who underwent CCTA screening. They used a short follow-up period and analyzed the results in the context of the presence of CAC as opposed to CCTA findings. Therefore, the conclusion of the meta-analysis by Waugh et al [23]—CCTA screening is not effective—was based on the finding that the risk of heart disease was not elevated in people undergoing a CAC assessment via CCTA, as opposed to an assessment of the prophylactic benefits of CCTA itself. Further, since the measurement of CAC is regarded as a reliable method for CVD risk assessment, a study claimed that CCTA should be introduced for the screening of asymptomatic individuals [24]. However, other studies claim that CCTA is cost-ineffective, although these admit that CAC, when observed via CCTA, is a better predictor of CVD than the FRS [25]. We supplemented these studies by comparing groups that underwent CCTA with those that did not undergo CCTA.

McEvoy et al [26] examined the differences in the incidence of coronary artery disease between CCTA and comparison groups after a fixed follow-up period. The authors matched the propensity scores of 1000 individuals who underwent CCTA for a health checkup with those of 1000 individuals who did not undergo CCTA (ie, the comparison group) and compared the incidence of coronary artery disease at the 90-day and 18-month follow-ups. The study reported that CCTA-based screening was significantly associated with an increased rate of invasive tests and medication use but was not associated with the incidence of coronary artery disease, concluding that CCTA is not recommended for screening purposes. However, the study was limited by the small number of cases and the short follow-up periods.

Our study presents reliable evidence about CCTA, which was obtained by performing large-scale propensity score matching and using EHR and health checkup questionnaire responses from OMOP-CDM data. We studied a large study sample over a longer study period than those used by previous studies. Although past studies used either 90-day follow-ups or 18-month follow-ups, we observed the patients from 60 days after the index date to 5 years after the index date to analyze the CVD HRs in relation to CCTA. Moreover, while previous studies had approximately 1000 patients in both the target group and comparison group, we included 8787 patients in each group. The data were also standardized, which enabled us to perform an efficient analysis across organizations and use the same analysis codes. Future studies can investigate the effects of CCTA and CVD in larger populations over long follow-up periods, in collaboration with organizations that convert health questionnaire data into the common data model format.

We also stratified the population into high-risk and low-risk groups based on the ASCVD risk score and FRS. Even in the high-risk group, CCTA screening did not have a significant effect (ASCVD risk score: calibration *P*=.39; FRS: calibration *P*=.13) on the prevention of CVD.

Based on the changes in risk scores, a significant difference was observed between the CCTA and comparison groups after 2 years (change in ASCVD risk scores: *P*=.03; change in FRSs: *P*=.02). However, this difference was not significant after 5 years (change in ASCVD risk score: *P*=.92; change in FRSs: *P*=.85). We speculate that patients are motivated to manage their risk score factors for a brief period immediately after the CCTA test; however, the significance decreases over long periods.

### Conclusions

Through a retrospective cohort study that was conducted over a 5-year period, we found that CCTA had no significant preventive effect on future CVDs. We also demonstrated the potential of converting health checkup data into OMOP-CDM data and integrating such data into common data model–based EHR data for research targeting the health checkup population. Although we examined the outcomes of CVDs after CCTA, future studies could examine patients’ health behaviors following CCTA. It is expected that the use of common data model data will be expanded to multicenter studies.

## Appendix

Multimedia Appendix 1 Supplementary material.

## Abbreviations

ACC/AHA: American College of Cardiology/American Heart Association
ASCVD: atherosclerotic cardiovascular disease
CAC: coronary artery calcium
CCTA: coronary artery computed tomography angiography
CHA2DS2-VASc: Congestive Heart Failure, Hypertension, Age≥75 (Doubled), Diabetes, Stroke (Doubled), Vascular Disease, Age 65 to 74, and Sex Category (Female)
CHADS2: Congestive Heart Failure, Hypertension, Age, Diabetes, Previous Stroke/Transient Ischemic Attack (2 points)
CVD: cardio-cerebrovascular disease
EHR: electronic health record
FRS: Framingham risk score
HR: hazard ratio
ICD-10: International Classification of Diseases, 10th Revision
IRB: Institutional Review Board
OHDSI: Observational Health Data Sciences and Informatics
OMOP-CDM: Observational Medical Outcomes Partnership Common Data Model
SNUBH: Seoul National University Bundang Hospital

## Data Availability

Common data model data are designed to support a distributed research network. Thus, access to the data is restricted on internal private networks, and the data are not publicly available.
