# Supplementary material for: Coronary Artery Computed Tomography Angiography for Preventing Cardio-Cerebrovascular Disease: Observational Cohort Study Using the Observational Health Data Sciences and Informatics’ Common Data Model
Source: JMIR Med Inform. 2022 Oct 13;10(10):e41503. doi: 10.2196/41503 (PMC9614618; doi:10.2196/41503)
Supplement: Multimedia Appendix 1 [file medinform_v10i10e41503_app1.docx]

**Supplementary material**

**Figure S1.** Distribution of propensity scores(PS) before and after PS matching for each subgroup (a) Before and (b) after PS matching of high-risk group of Framingham risk score, (c) Before and (d) after PS matching of low-risk group of Framingham risk score, (e) Before and (f) after PS matching of high-risk group of ACC/AHA ASCVD risk score and (g) Before and (h) after PS matching of low-risk group of ACC/AHA ASCVD risk score.

(a)

**
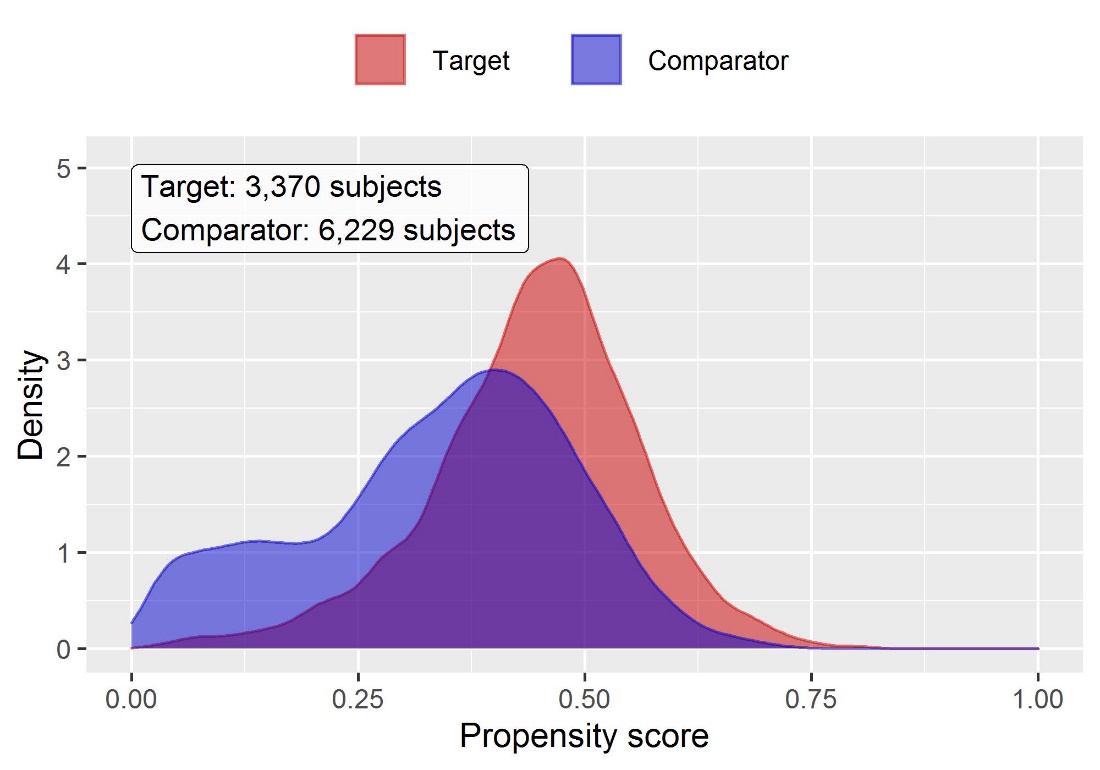
**

(b)

**
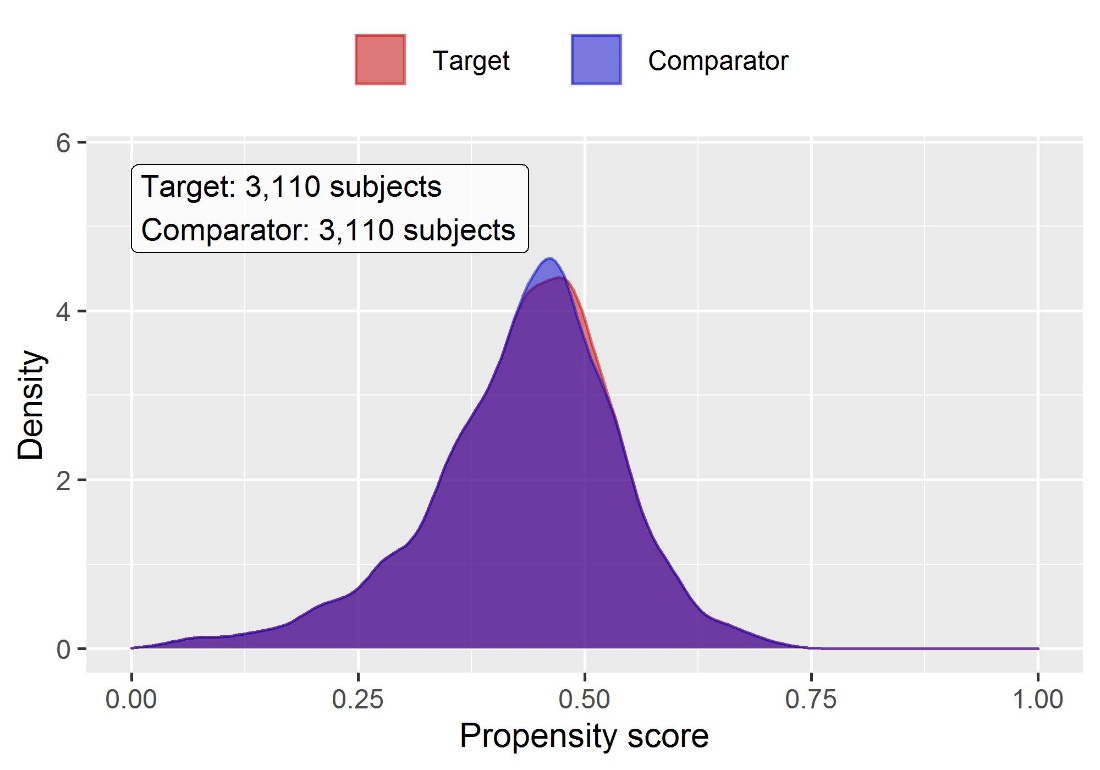
**

(c)


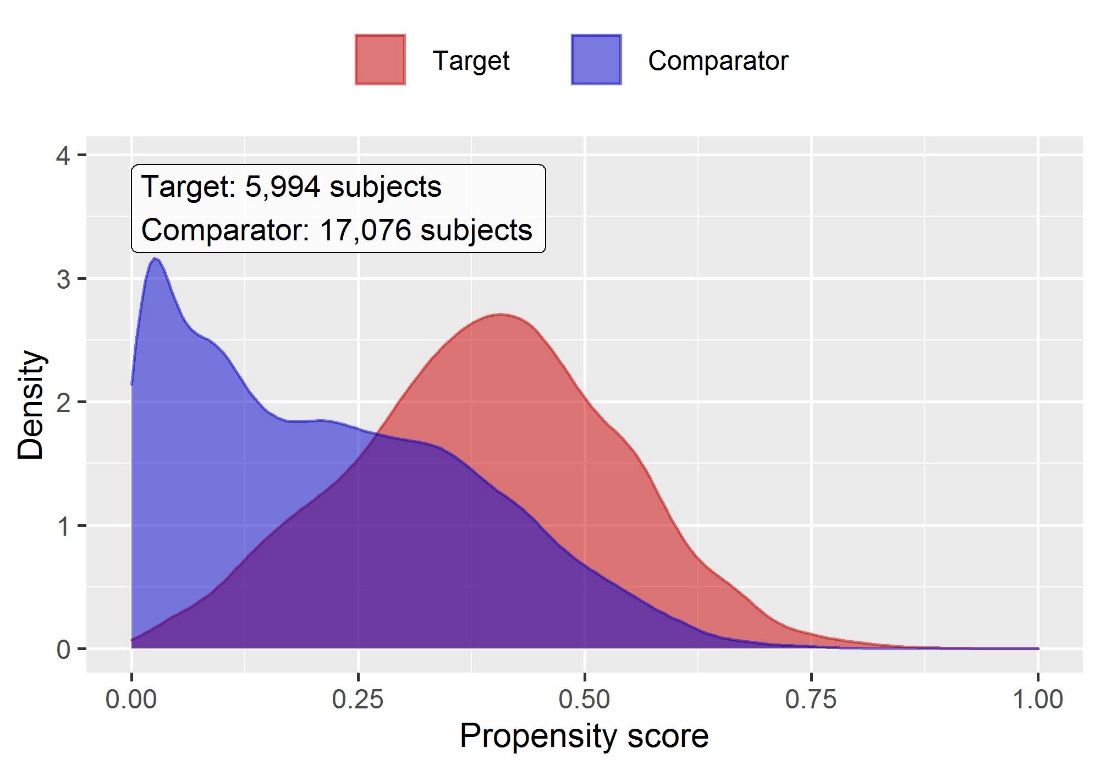


(d)


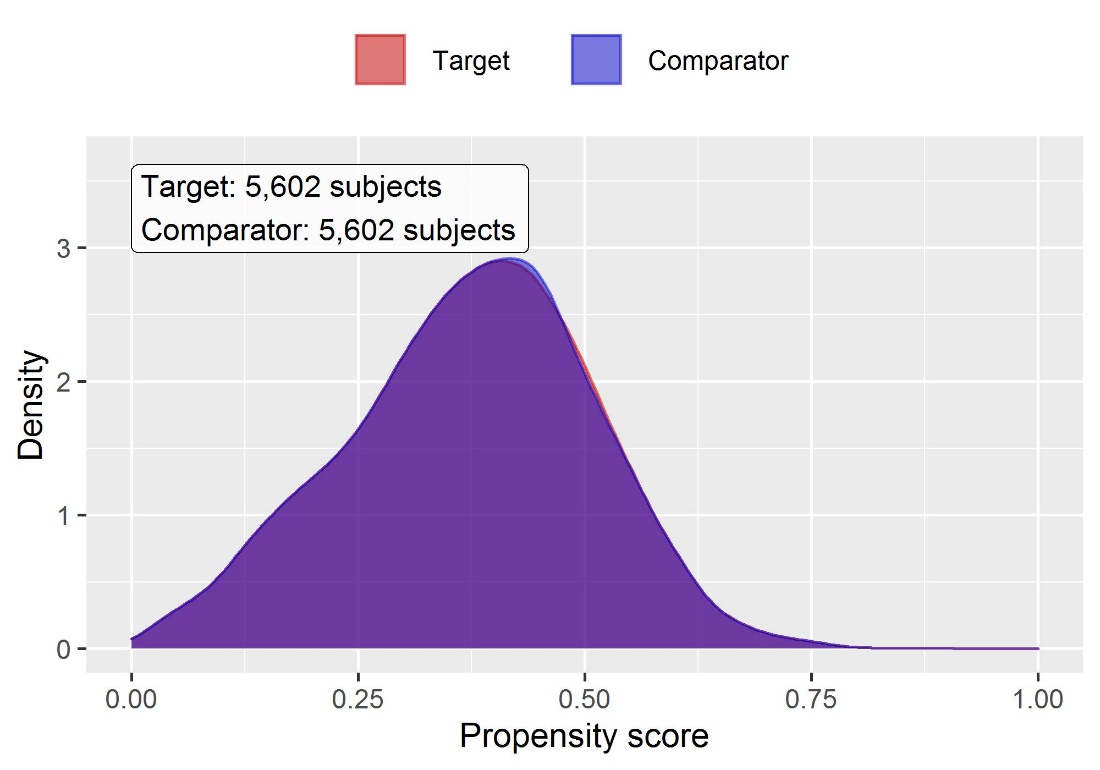


(e)


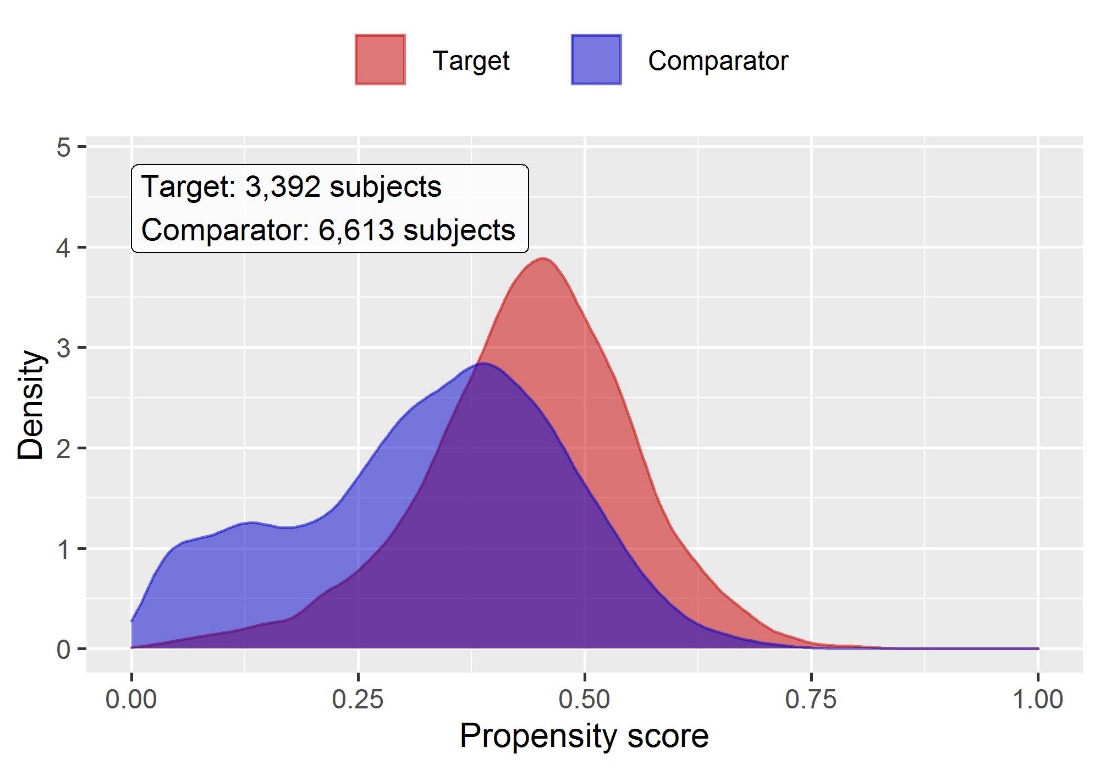


(f)


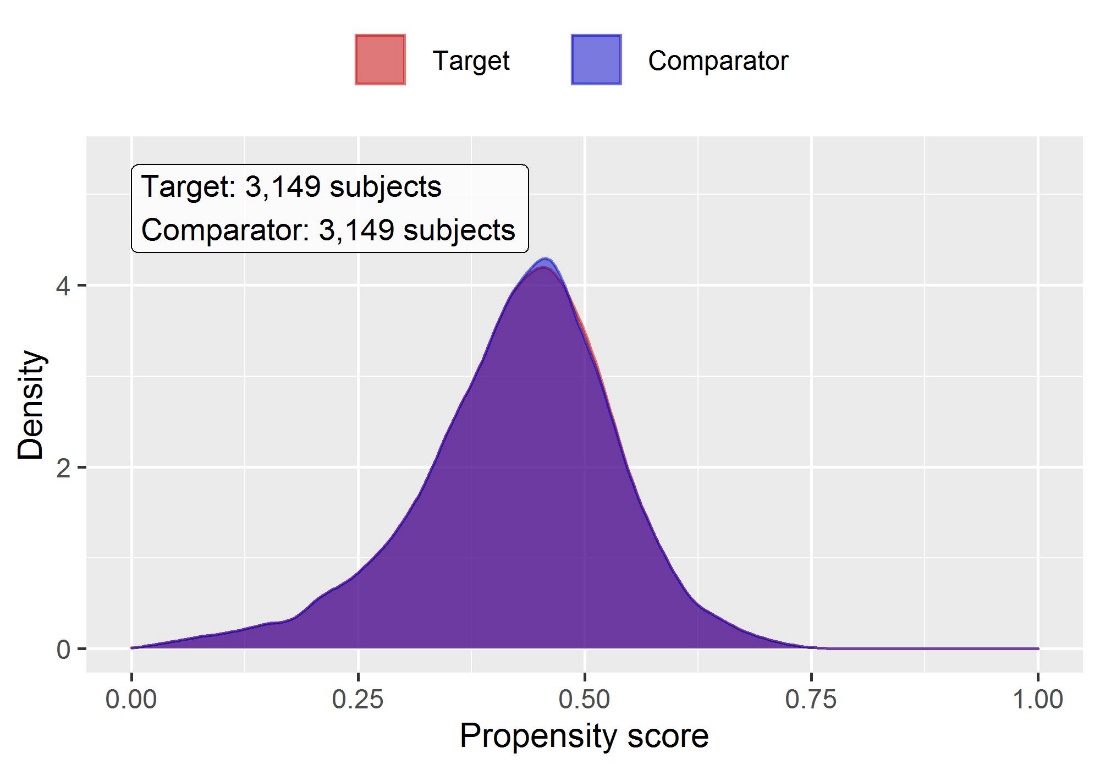


(g)


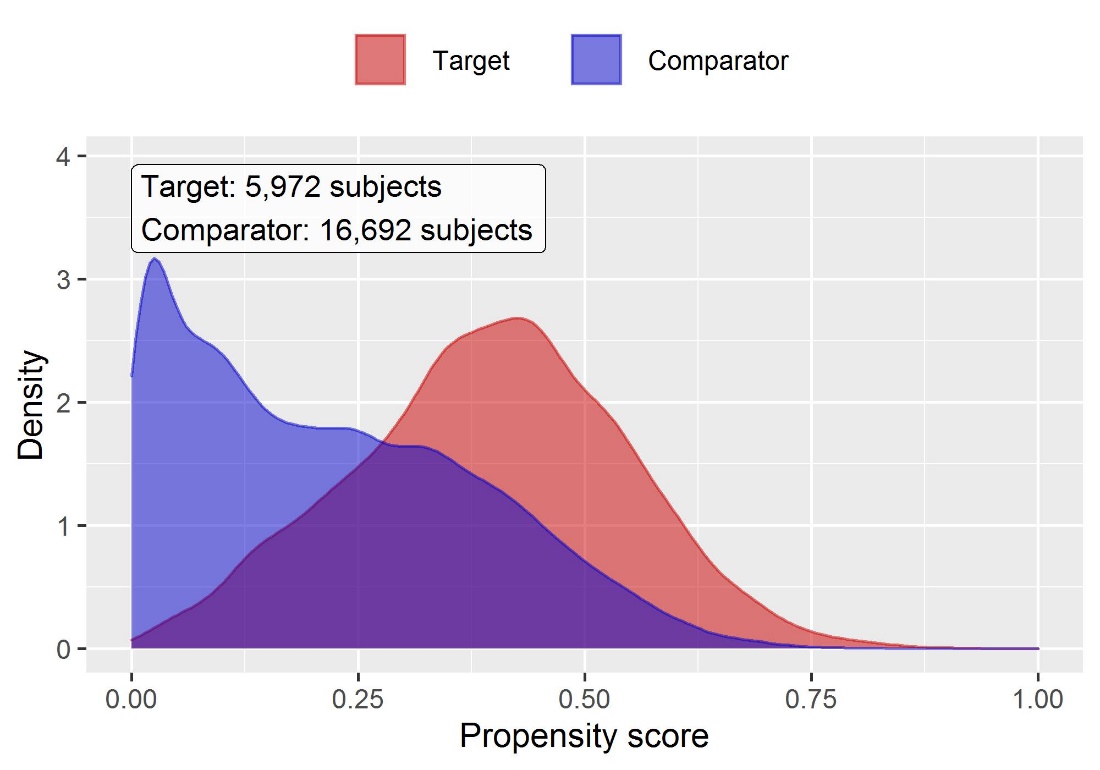


(h)


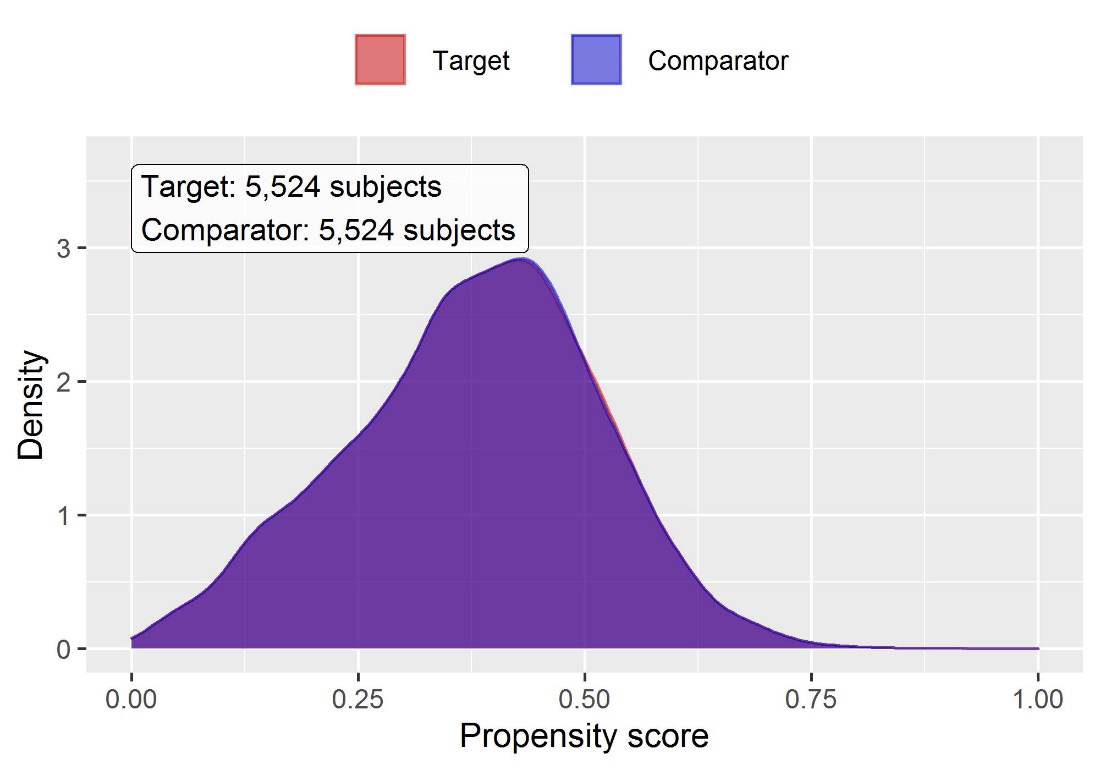


**Figure S2.** Standardized difference of mean between groups of covariates before and after propensity score matching of each subgroup (a) high-risk group of Framingham risk score, (b) low-risk group of Framingham risk score, (c) high-risk group of ACC/AHA ASCVD risk score and (d) low-risk group of ACC/AHA ASCVD risk score

(a)


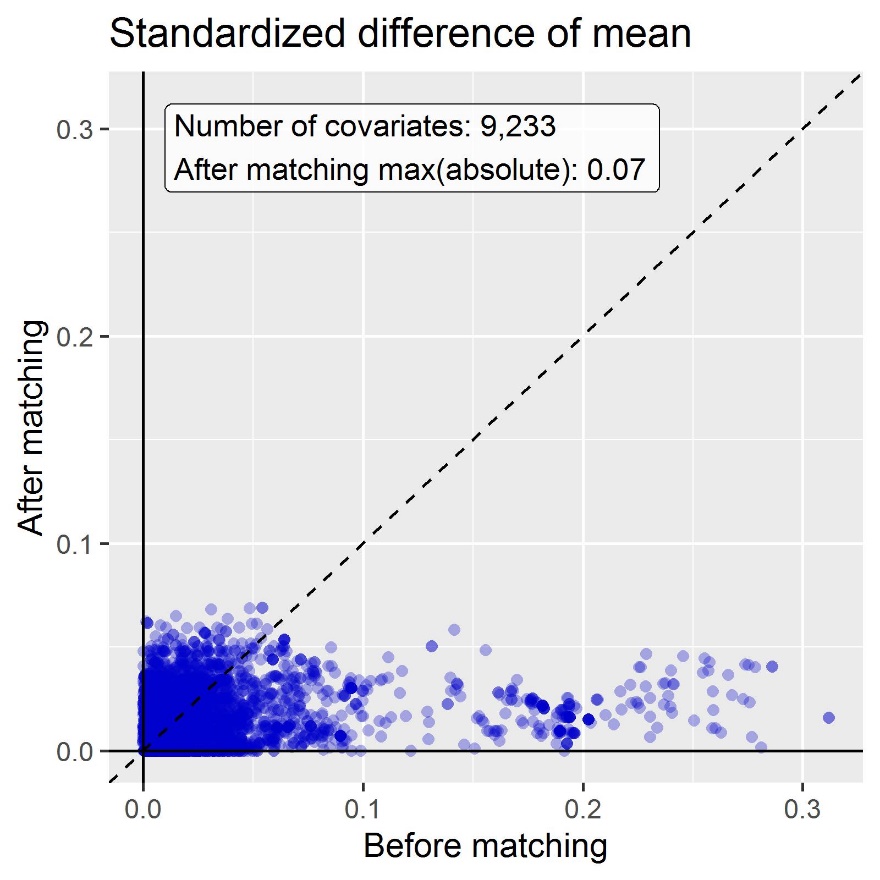


(b)


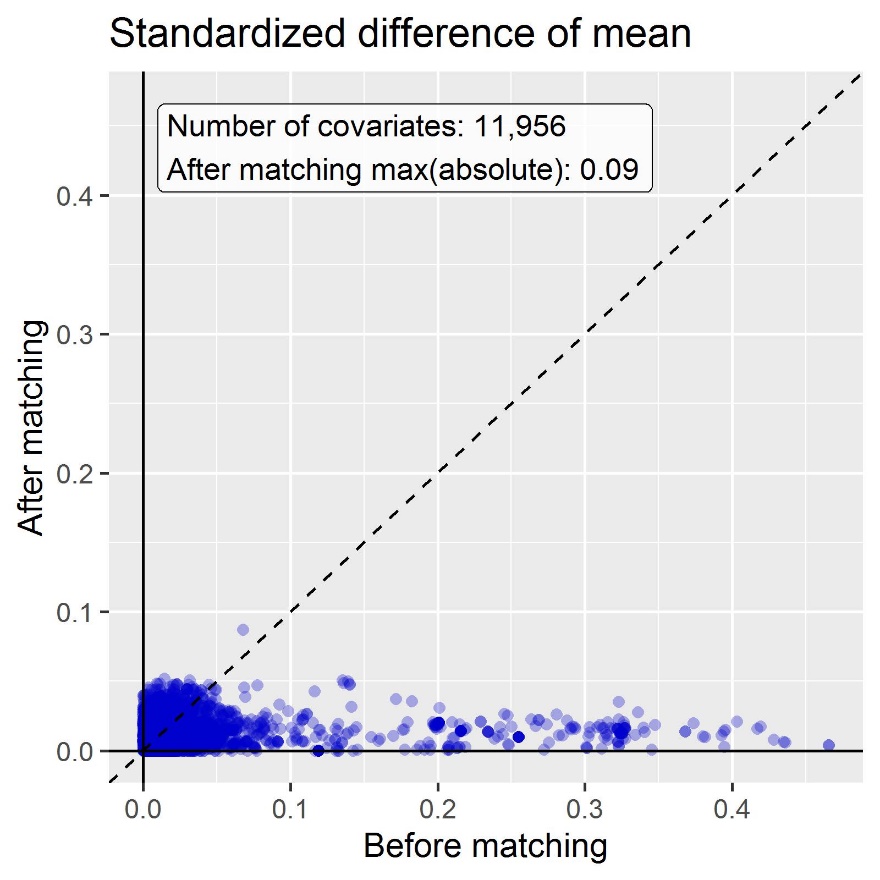


(c)


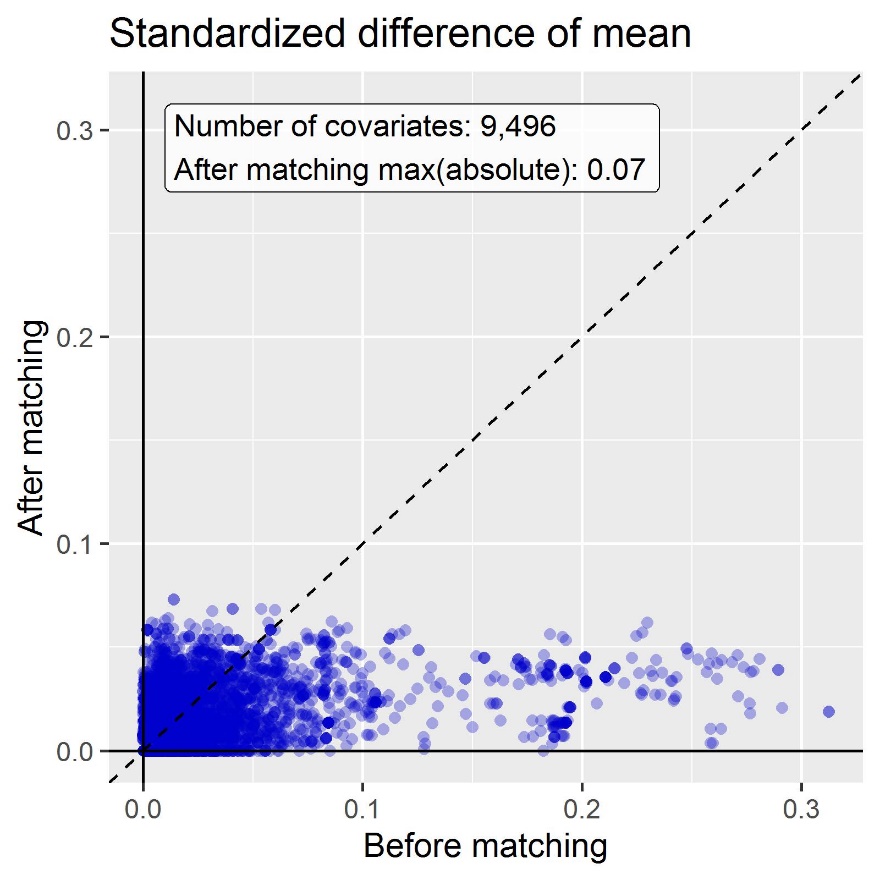


(d)


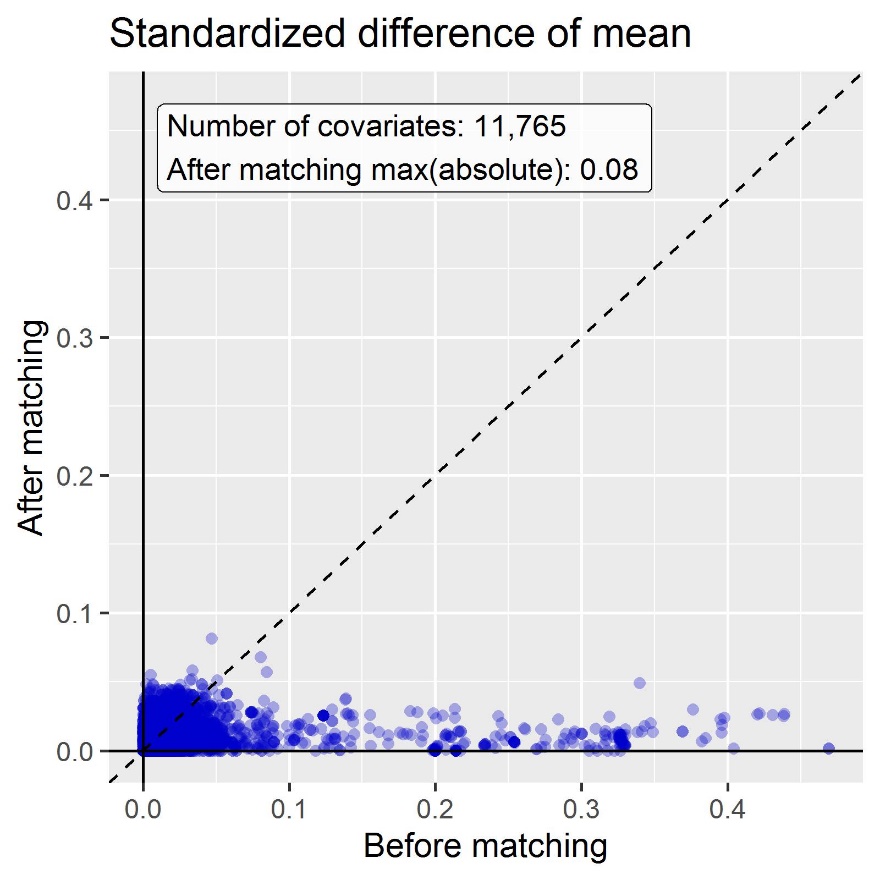


**Figure S3.** Kaplan Meier curve plot and rejection area plot with negative outcome controls in each subgroup (a), (b) high-risk group of Framingham risk score, (c), (d) low-risk group of Framingham risk score, (e), (f) high-risk group of ACC/AHA ASCVD risk score and (g), (h) low-risk group of ACC/AHA ASCVD risk score

(a)


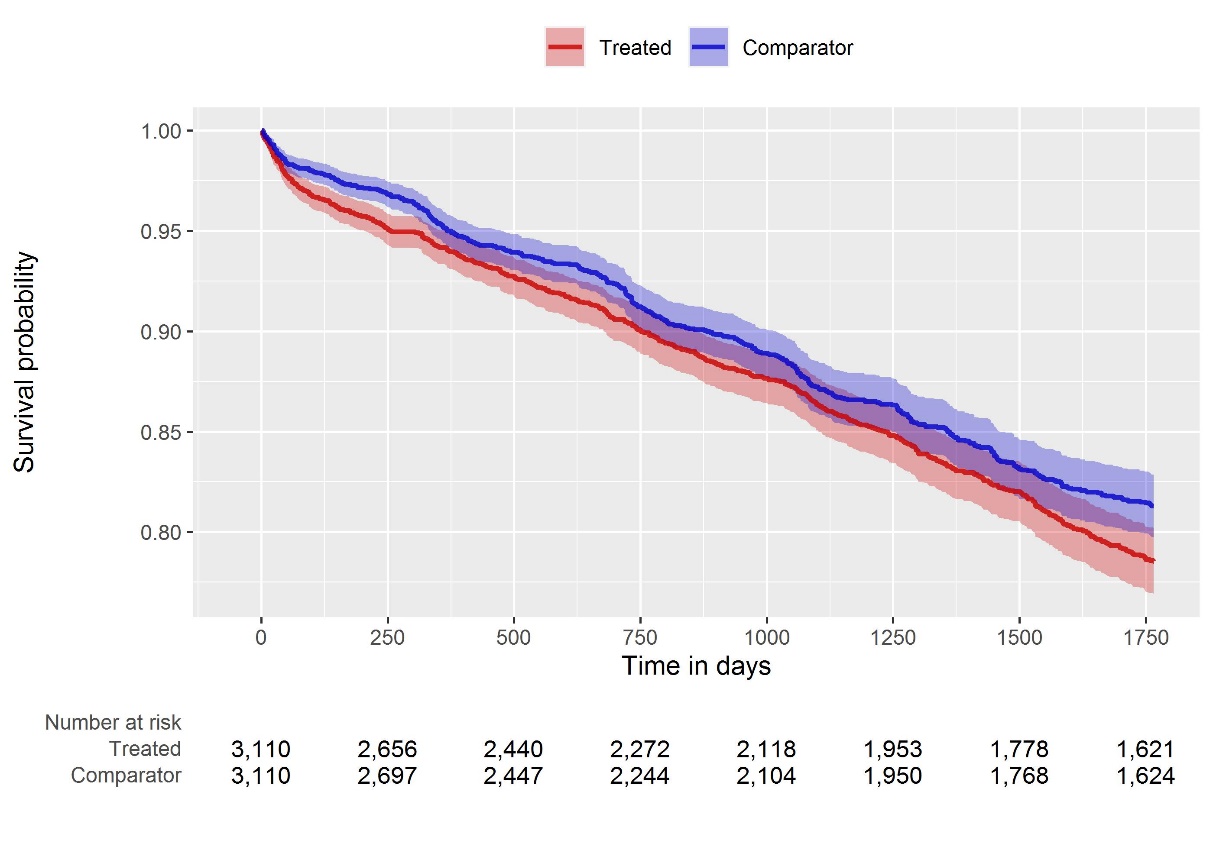


(b)


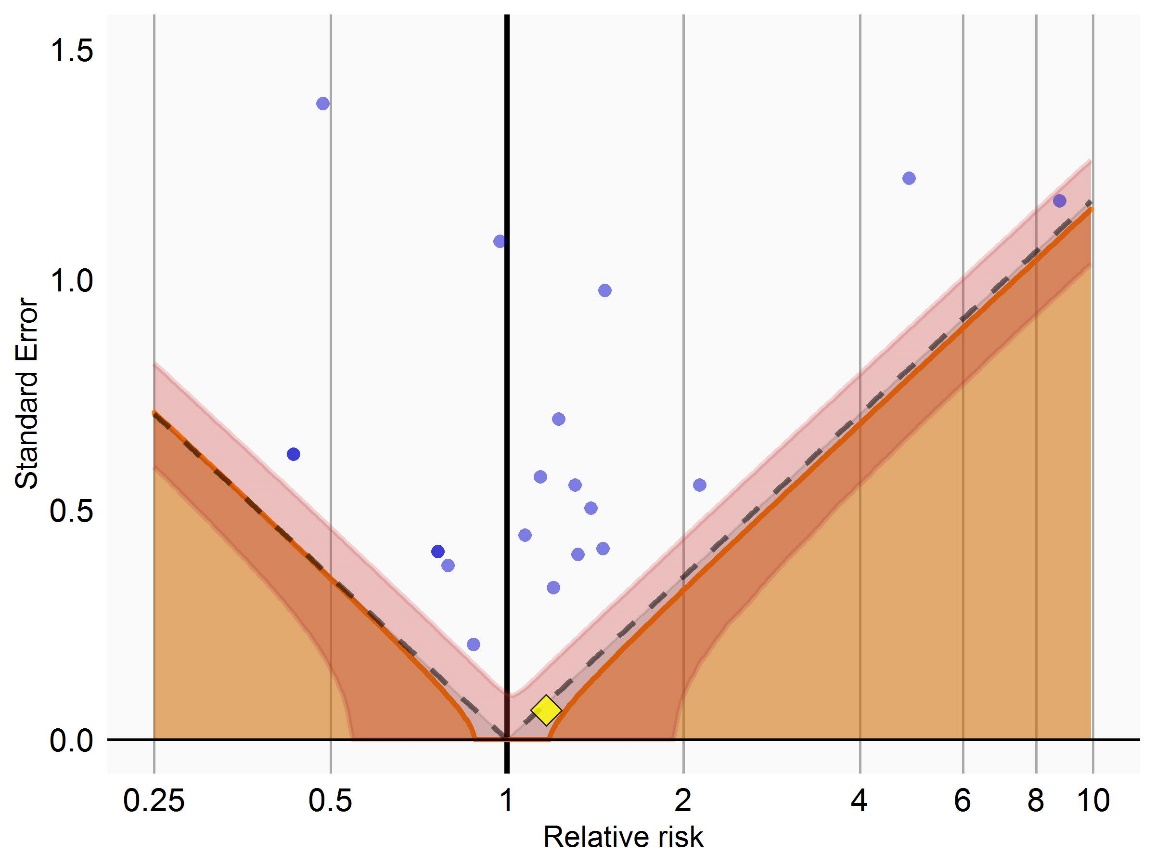


(c)


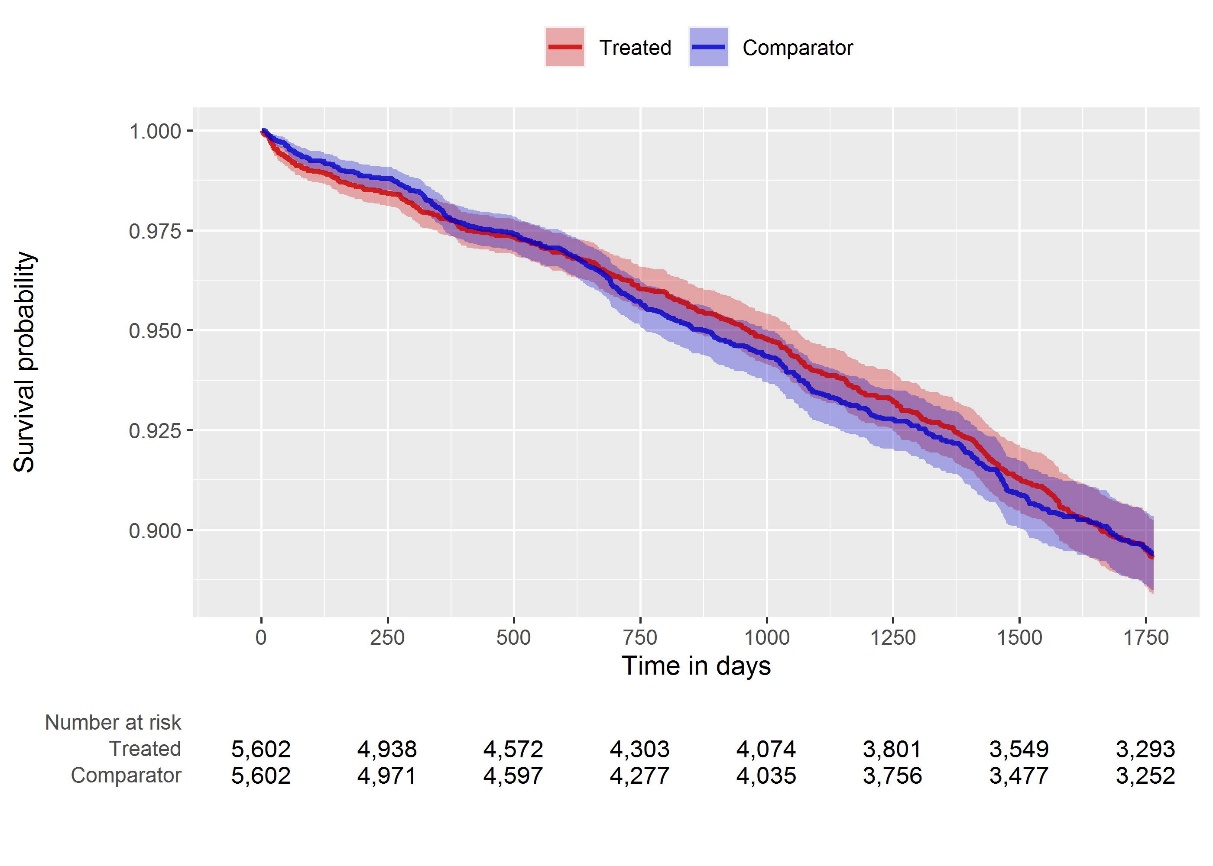


(d)


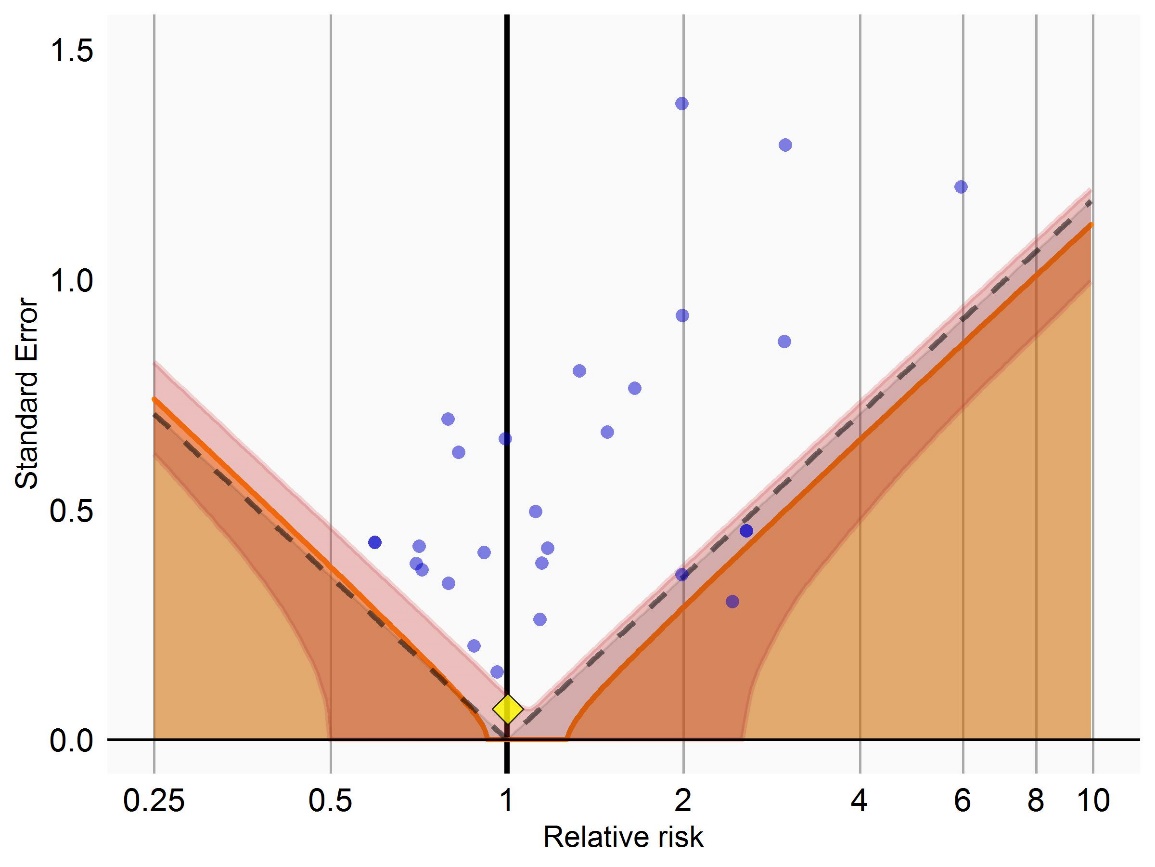


(e)


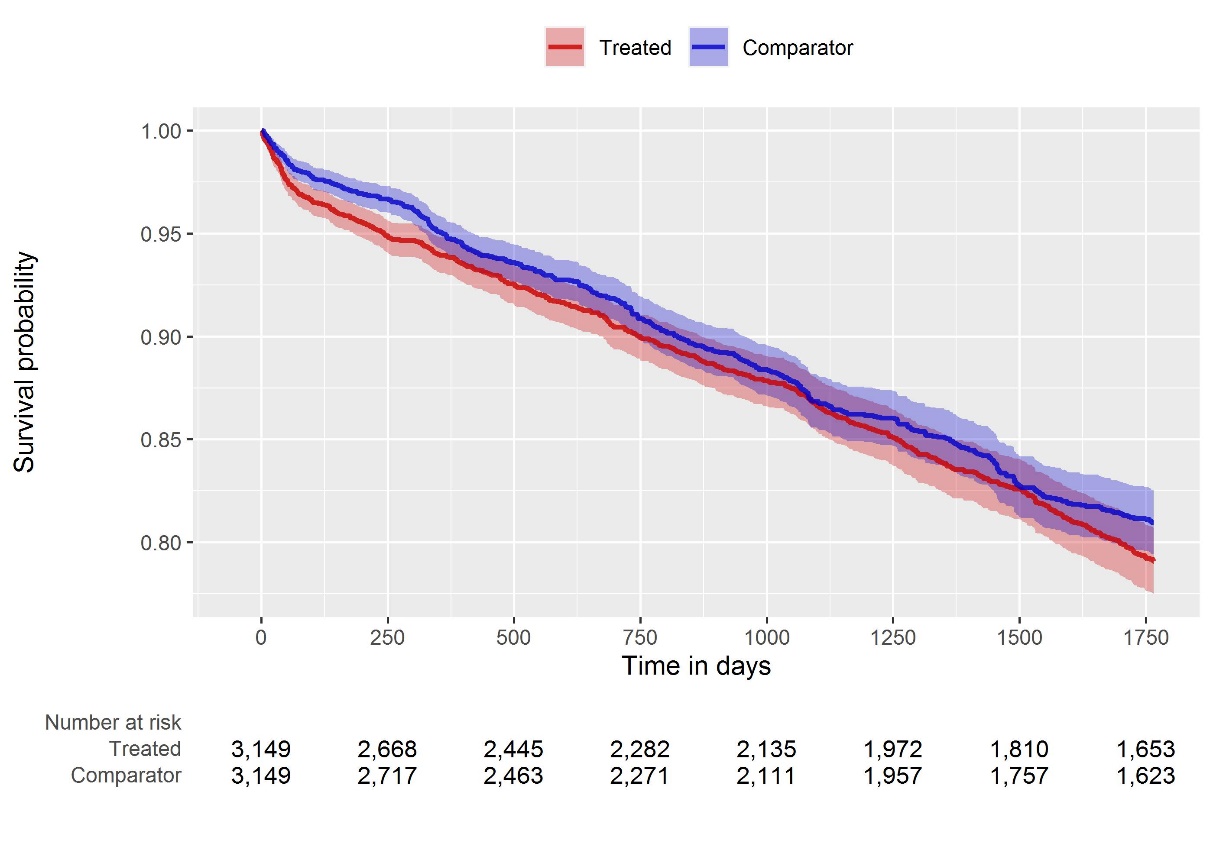


(f)


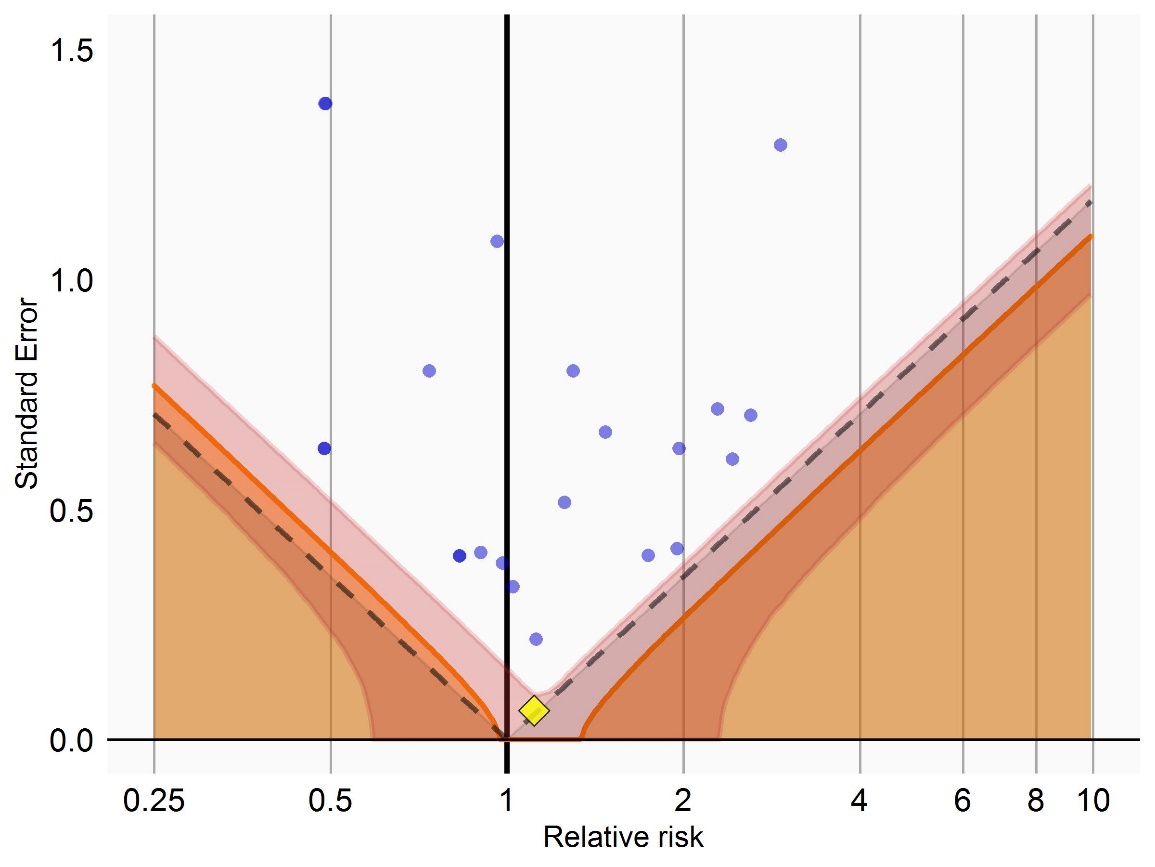


(g)


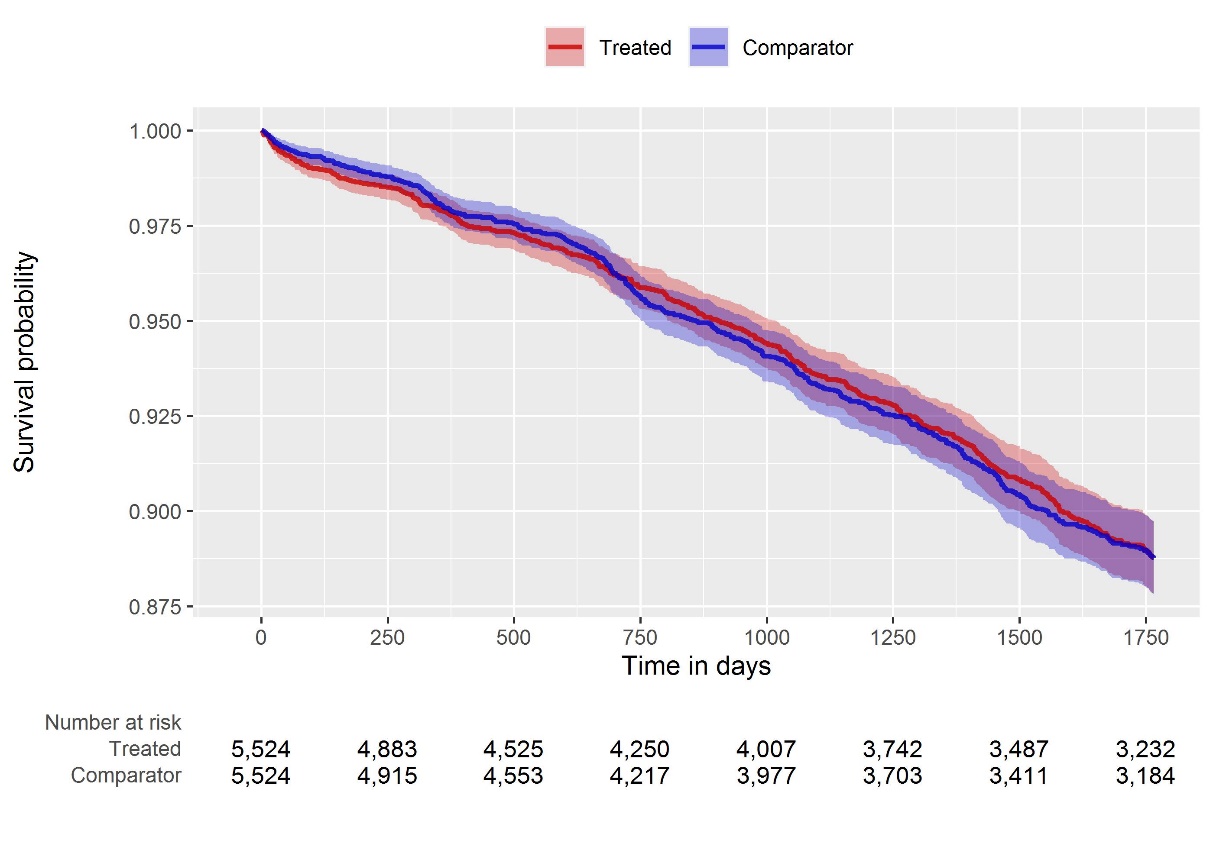


(h)


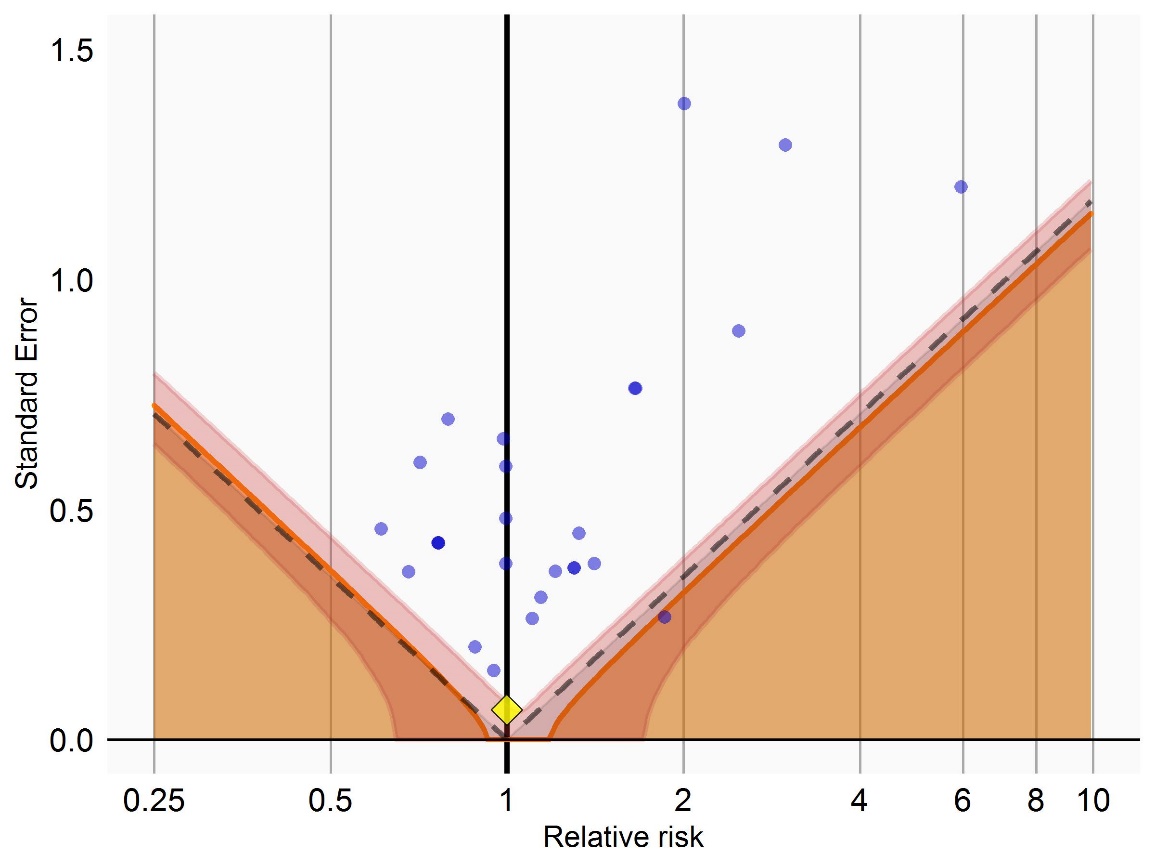


**Table S1.** negative control outcome

| **Outcome Id** | **Outcome Name** |
| --- | --- |
| 434165 | Abnormal cervical smear |
| 436409 | Abnormal pupil |
| 199192 | Abrasion and/or friction burn of trunk without infection |
| 4088290 | Absence of breast |
| 4092879 | Absent kidney |
| 44783954 | Acid reflux |
| 75911 | Acquired hallux valgus |
| 137951 | Acquired keratoderma |
| 77965 | Acquired trigger finger |
| 376707 | Acute conjunctivitis |
| 4103640 | Amputated foot |
| 73241 | Anal and rectal polyp |
| 133655 | Burn of forearm |
| 73560 | Calcaneal spur |
| 434327 | Cannabis abuse |
| 4213540 | Cervical somatic dysfunction |
| 140842 | Changes in skin texture |
| 81378 | Chondromalacia of patella |
| 432303 | Cocaine abuse |
| 4201390 | Colostomy present |
| 46269889 | Complication due to Crohn's disease |
| 134438 | Contact dermatitis |
| 78619 | Contusion of knee |
| 76786 | Derangement of knee |
| 4115402 | Difficulty sleeping |
| 45757370 | Disproportion of reconstructed breast |
| 433111 | Effects of hunger |
| 433527 | Endometriosis |
| 4170770 | Epidermoid cyst |
| 4092896 | Feces contents abnormal |
| 259995 | Foreign body in orifice |
| 40481632 | Ganglion cyst |
| 4166231 | Genetic predisposition |
| 433577 | Hammer toe |
| 440329 | Herpes zoster without complication |
| 4012570 | High risk sexual behavior |
| 4012934 | Homocystinuria |
| 441788 | Human papilloma virus infection |
| 4201717 | Ileostomy present |
| 374375 | Impacted cerumen |
| 4344500 | Impingement syndrome of shoulder region |
| 139099 | Ingrowing nail |
| 444132 | Injury of knee |
| 196168 | Irregular periods |
| 432593 | Kwashiorkor |
| 434203 | Late effect of contusion |
| 438329 | Late effect of motor vehicle accident |
| 195873 | Leukorrhea |
| 4083487 | Macular drusen |
| 4103703 | Melena |
| 377572 | Noise effects on inner ear |
| 40480893 | Nonspecific tuberculin test reaction |
| 136368 | Non-toxic multinodular goiter |
| 140648 | Onychomycosis due to dermatophyte |
| 438130 | Opioid abuse |
| 4091513 | Passing flatus |
| 4202045 | Postviral fatigue syndrome |
| 373478 | Presbyopia |
| 46286594 | Problem related to lifestyle |
| 439790 | Psychalgia |
| 81634 | Ptotic breast |
| 380706 | Regular astigmatism |
| 141932 | Senile hyperkeratosis |
| 36713918 | Somatic dysfunction of lumbar region |
| 443172 | Splinter of face, without major open wound |
| 81151 | Sprain of ankle |
| 72748 | Strain of rotator cuff capsule |
| 378427 | Tear film insufficiency |
| 194083 | Vaginitis and vulvovaginitis |
| 140641 | Verruca vulgaris |
| 4115367 | Wrist joint pain |
| 440193 | Wristdrop |
